# Four novel variants identified in the *ACADVL* gene causing very-long-chain acyl-coenzyme A dehydrogenase deficiency in four unrelated Chinese families

**DOI:** 10.3389/fgene.2024.1433160

**Published:** 2024-08-12

**Authors:** Lulu Li, Yue Tang, Jinqi Zhao, Lifei Gong, Nan Yang, Shunan Wang, Haihe Yang, Yuanyuan Kong

**Affiliations:** Department of Newborn Screening Center, Beijing Obstetrics and Gynecology Hospital, Capital Medical University, Beijing Maternal and Child Healthcare Hospital, Beijing, China

**Keywords:** very-long-chain acyl-CoA dehydrogenase deficiency, VLCADD, *ACADVL*, genetic testing, rare disease

## Abstract

**Background**: The biochemical and genetic characteristics of four very-long-chain acyl-coenzyme A dehydrogenase deficiency (VLCADD) patients, clarifying their pathogenic genetic factors and evaluating the application value of genetic diagnosis in the early diagnosis of VLCADD, are reported and discussed in this article.

**Methods**: Patients underwent blood tandem mass spectrometry (MS/MS), urine gas chromatography (GC/MS), and high-throughput sequencing technology. New variants were analyzed for pathogenicity using bioinformatics software. Swiss-PdbViewer software was used to predict the effect of variants on the structure of the very-long-chain acyl-CoA dehydrogenase (VLCAD) protein.

**Result**: A total of four VLCADD patients were diagnosed. They revealed elevated levels of C14, C14:1, C14:2, C14:1/C2, C14:1/C10, and C14:1/C12:1. Two patients were early-onset neonatal cases and died during infancy and the neonatal period, respectively. Seven kinds of variants were detected, including four novel variants. Bioinformatics software revealed that the variants were harmful, and the Swiss-PdbViewer results suggest that variation affects protein conformation.

**Conclusion**: This study identified four novel *ACADVL* gene variants. These findings contribute to the understanding of the genetic basis and pathogenesis of VLCADD. Meanwhile, the study enriches the genetic mutation spectrum and the correlation between genotypes and phenotypes of VLCADD, indicating that genetic diagnosis plays an essential role in the early diagnosis and treatment of VLCADD.

## 1 Introduction

Very-long-chain acyl-CoA dehydrogenase deficiency (VLCADD; OMIM 201475) is a rare autosomal recessive inherited disorder of fatty acid oxidation. It is caused by a congenital defect in the encoding gene, *ACADVL* (OMIM 609575), of very-long-chain acyl-CoA dehydrogenase (VLCAD), which leads to long-chain fatty acid oxidation disorders (Aoyama et al., 1993; Strauss et al., 1995). VLCAD is the first key enzyme in mitochondrial fatty acid β oxidation on the inner mitochondrial membrane. VLCAD undergoes catalytic dehydrogenation of acyl CoA containing 14–18 carbons, and fatty acid β oxidation is completed under the catalysis of multiple enzymes. Acetyl-CoA is produced at the same time, and it participates in the tricarboxylic acid cycle for energy supply. It can also form ketone bodies in the liver, generating energy during exercise, hunger, stress, and other conditions. VLCAD provides energy sources for essential organs such as the skeletal muscles, heart, and liver (El-Gharbawy and Vockley, 2018). VLCAD deficiency results in impaired fatty acid β oxidation, energy supply disorders, and accumulation of long-chain acylcarnitines, thus producing toxic effects on the heart, liver, skeletal muscle, and other organs, causing a series of related clinical symptoms and signs, which can lead to sudden death in severe cases (Vianey-Saban et al., 1998).

VLCADD was first reported by Bertrand et al. (1993), with apparent ethnic and regional differences. The prevalence rate of VLCADD in Europe and America is 1/100,000–1/30,000 (Rovelli et al., 2019), and the prevalence rate of VLCADD in Asia ranges from 1/1400,000 to 1/380,000 (Shibata et al., 2018). The disease is rare in China, with a population prevalence of 1/236,655–1/70,424 (Division of Biochemistry and Metabolism et al., 2022). The clinical manifestations of VLCADD are complex, with strong (substantial) phenotypic heterogeneity, and can occur from newborns to adults. According to the age of onset and clinical manifestations, VLCADD can be divided into three types (Arnold et al., 2009; Division of Biochemistry and Metabolism et al., 2022): 1) the early-onset form is the most severe and begins in infancy, and often has myocardial involvement. This type is dangerous and has a high mortality rate in children. It is characterized by hypoketotic hypoglycemia, feeding difficulties, respiratory distress, sudden neonatal death, hypertrophic and dilated cardiomyopathy, arrhythmias, pericardial effusion, and multi-organ failure; 2) the infantile form, also known as the hepatopathy form, has a late infantile or childhood onset. It is of moderate clinical severity and often presents with recurrent episodes of hypoketotic hypoglycemia and abnormal liver function. It usually manifests itself only after the first episode of infectious disease, followed by secondary episodes of hypoglycemia, rarely accompanied by myocardial damage; and 3) the late-onset form, also known as the myopathy type, occurs in adolescence or adulthood. The myopathy type is mild and has the best prognosis. Generally, there are no myocardial disease and hypoglycemia, and the main manifestations are rhabdomyolysis and myoglobinuria after exercise, infection, or starvation.

VLCADD patients are given high-carbohydrate and low-fat diets, limit the intake of long-chain fatty acids, supplement medium-chain triglycerides, avoid fasting and hunger, prevent fat decomposition, ensure that their body has enough calories and energy, maintain stable blood sugar levels, and ensure symptomatic treatment and treatment of complications (Strauss et al., 1995; Nurjanah et al., 2023; Al Bandari et al., 2024).

At present, tandem mass spectrometry (MS) is often used to detect the elevation of C14:1 as a specific diagnostic indicator (Wood et al., 2001; Zytkovicz et al., 2001). A growing number of children with VLCADD could be found in neonatal screening with the wide application of tandem mass spectrometry. The false-positive rate of MS/MS is very high, and it is usually difficult to distinguish true positives, heterozygous carriers, and false positives, which indicates the limitations through biochemical testing (Boneh et al., 2006; Schymik et al., 2006; Burrage et al., 2016). Sometimes, diagnosis may be missed if future examination is not conducted.

Clinical characteristics, biochemical tests, and genetic results of four patients with VLCADD were analyzed in this study at the Beijing Neonatal Disease Screening Center to explore the genetic etiology, genotype, and phenotype correlation of VLCADD in order to improve the understanding, diagnosis, and treatment level of this disease.

## 2 Methods and materials

### 2.1 Medical records and ethical sight

Case 1: a female child, who was 21 days old at first diagnosis, was admitted to the Beijing Neonatal Disease Screening Center due to high C14:1 levels observed by tandem mass spectrometry. The child was delivered via cesarean section at 41 weeks, with height of 50 cm and weight of 3,300 g. Her mother had no special conditions during pregnancy. The child was admitted to the ICU of the Pediatric Research Institute for treatment due to crying and frequent vomiting of milk at 3 months and died 7 days later.

Case 2: a female child was born through vaginal delivery, with height of 50 cm and weight of 3,400 g. The child died suddenly 2 days after birth due to hypoglycemia, acidosis, and abnormal liver and kidney function. Her peripheral blood was sent to the Beijing Neonatal Disease Screening Center for tandem mass spectrometry screening and genetic detection.

Case 3: a male child, 14 days old at first diagnosis, was admitted to the Beijing Neonatal Disease Screening Center due to high C14:1 levels observed by tandem mass spectrometry. The child was 38 weeks and born through vaginal delivery, with height of 49 cm and weight of 3,130 g, good nursing after birth, normal complexion, and sensitive response. In the mother’s first pregnancy, the fetus appeared in fetal arrest due to trisomy 7.

Case 4: a male child, 17 days old at first diagnosis, was admitted to the Beijing Neonatal Disease Screening Center due to high C14:1 levels observed by tandem mass spectrometry. The child was 40 weeks and born through vaginal delivery, with height of 53 cm and weight of 3,520 g, good nursing after birth, normal complexion, and sensitive response.

The parents of all the mentioned patients had normal phenotypes, no consanguineous marriage, and no family history of genetic or infectious disease. The Beijing Obstetrics Gynecology Hospital of Capital Medical University Ethics Committee approved this study (2022-KY-087-01), and all family members (or guardians) provided informed consent.

### 2.2 Blood tandem mass spectrometry

After 72 h of birth with a fully breastfed newborn, at least eight times, the heel blood spots were collected for tandem mass spectrometry detection. A 3-mm diameter of blood spots was taken from each specimen and the TQD tandem mass spectrometer (TQ Acquisition Mass Spectrometer, Waters, United States) was used. Non-derivative screening kits (non-derivative MSMS Kit, PerkinElmer, United States) were used to detect amino acids and acylcarnitine in the samples.

### 2.3 Gas chromatography–mass spectrometry

A measure of 5–10 mL of fresh urine was collected from the patients, and urine was treated by removing urea, adding an internal standard, removing protein, vacuum drying, performing trimethylsilane derivatization, *etc*. The organic acids, amino acids, and other components in urine were analyzed using gas chromatography–mass spectrometry (Shimadzu GCMS-QP2010), and the detection peaks were qualitatively and quantitatively analyzed.

### 2.4 High-throughput sequencing and bioinformatics analysis

Libraries of genomic DNA samples were sequenced on the AmCareSeq-2000 sequencer (AmCare Genomics Lab, Guangzhou, China). The average coverage depth was 100–200 ×, with >96% of the target regions covered by at least 20 reads. The experimental procedures were as follows: DNA extraction, genomic library preparation, hybridization capture, computer sequencing, data filtering, and analysis. The variants were cross-checked in the Human Gene Mutation Database (HGMD; http://www.hgmd.cf.ac.uk/ac/index.php) to know whether the identified variants are novel or already reported.

### 2.5 Primer designing and pathogenic variant confirmation

The results of candidate pathogenic variants screened by high-throughput sequencing showed that the ACADVL (NM_000018.4) gene sequence was obtained from the University of California Santa Cruz (UCSC) (http://genome.ucsc.edu). The standard procedures involved performing PCR amplification using the Primer 5.0 design tool to design specific PCR primers (Supplementary Table S1); the PCR amplification was performed according to standard procedures. The PCR products were purified and processed through DNA sequencing. The sequencing results were analyzed using CodonCode Aligner software, along with parental origin verification.

### 2.6 Predictive analysis of pathogenicity

Functional prediction of missense mutant loci was performed using Sorting Intolerant From Tolerant (SIFT; http://www.sift.jcvi.org/), PolyPhen-2 (http://genetics.bwh.harvard.edu/pph2), MutationTaster (http://www.mutationtaster.org/), and REVEL (https://sites.google.com/site/revelgenomics/). Variant frequencies were determined in the 1000 Genomes Project, ExAC (http://exac.broadinstitute.org/), and gnomAD (http://gnomad-sg.org/) database. Finally, the American College of Medical Genetics and Genomics (ACMG) 2019 guidelines were used to interpret variants.

Swiss-Pdb viewer software was used to predict the evaluation of the crystal structure of the mutant proteins. The protein structures of VLCAD (PDB ID: 2UXW) were acquired from the PDB and combined with Swiss-Pdb viewer 4.1.0 software to visualize the protein structures and predict the effect of the variant sites on the tertiary protein structures.

## 3 Results

### 3.1 Clinical characteristics

This study reported four VLCADD cases: two males and two females; all four cases were born at full term. Case 1 appeared to have an acute metabolic crisis 3 months after birth and died in infancy, and case 2 developed an acute metabolic crisis 2 days after birth and died in the neonatal period.

The two cases belong to the neonatal early-onset form (Table 1). Case 2 was the sample tested after the death of the child. Case 3, currently 4 months old, with height of 62 cm and weight of 7 kg, is able to lift and roll over, and has overall good development. There were no clinical symptoms such as hypoglycemia, cardiac abnormality, and liver enlargement. Milk powder rich in medium-chain fatty acids was used for feeding. Case 4, currently over 2 months old, with height of 60.5 cm and weight of 6 kg, has overall good development. There were no clinical symptoms such as hypoglycemia, cardiac abnormality, and liver enlargement. The current feeding method combined breast milk and milk powder rich in medium-chain fatty acids (Table 1).

**TABLE 1:** Clinical and genetic characteristics of patients recruited in this study.

Case	Gender	Age at first visit	Age at diagnosis	Age at onset	Clinical outcome	Clinical phenotype	Genetic characteristics						
							Gene	Zygote type	Allele origin	Variant location	Nucleotide (amino acid) change	Novel variant	ACMG
1	Female	21 d	25 d	3 m	Acute metabolic crisis 3 months after birth and died in infancy	Neonatal early-onset form	*ACADVL*	C-het	P	E4	c.218T>C (p.Glu73Ser)	Y	VUS (PM2+PP3+PM1+PP2)
									M	E13	c.1292A>G (p.Asp431Gly)	N	LP (PM1+PM2+PP3+PP2)
2	Female	—	After death	2 d	Acute metabolic crisis 2 days after birth and died in the neonatal period	Neonatal early-onset form	*ACADVL*	C-het	P	I13	c.1332 + 1G>C	Y	LP (PVS1+PM2)
									M	E9	c.862_870del (p.Phe288_Gly290del)	Y	LP (PM1+PM2+PM4)
3	Male	14 d	17 d	—	Currently asymptomatic, good growth and development, and Meiji formula feeding	Late-onset form	*ACADVL*	C-het	P	E14	c.1349G>A (p.Arg450His)	N	P (PM3+PM2+PM5+PM1+ +PP3+PP2)
									M	E7	c.553G>A (p.Gly185Ser)	N	P (PM3+PM2+PM5+PM1 +PP3+PP2)
4	Male	17 d	20 d	—	Currently asymptomatic, good growth and development, and breast milk and Meiji formula mixed feeding	Late-onset form	*ACADVL*	C-het	P	E7	c.480C>A (p.Tyr160*)	Y	LP (PVS1+PM2)
									M	E14	c.1349G>A (p.Arg450His)	N	P (PM3+PM2+PM5+PM1 +PP3+PP2)

d, day; m, month; Y, yes; N, no; hom, homozygote; C-het, compound heterozygote; P, paternal; M, maternal; E, exon; I, intron.

Blood tandem mass spectrometry screening of cases 1, 2, 3, and 4 showed significant elevation in C14, C14:1, C14:2, C14:1/C2, C14:1/C10, and C14:1/C12:1 levels (Table 2). The GC/MS results of case 3 showed that adipic acid, octametic acid, and 2-hydroxyadipic acid levels were slightly elevated; the GC/MS results of case 4 showed that glutaric acid, adipic acid, octametic acid, azelaic acid, lactic acid, pimelic acid, 2-hydroxysebacic acid, and 2-hydroxyadipic acid levels were significantly elevated (Supplementary Table S2).

**TABLE 2:** Test results of blood tandem mass spectrometry.

Case	Blood tandem mass spectrometry														
	C2	C4	C6	C8	C10	C12	C12:1	C14	C14:1	C14:2	C14:1/C2	C14:1/C10	C14:1/C12:1	C16	C18
1	0.94↓	0.05↓	0.01	0.02	0.07	0.13	0.19	0.62 ↑	1.3 ↑	0.29 ↑	26↑	18.57↑	32.5↑	1.16	0.7
2	10.64	0.21	0.04	0.06	0.19	0.4	0.04	3.62 ↑	2.03 ↑	0.13 ↑	9.67↑	10.68↑	10.68↑	9.73↑	2.49↑
3^a^	7.03	0.14	0.02	0.03	0.06	0.26	0.08	0.83 ↑	2.1 ↑	0.36 ↑	0.3↑	35↑	26.25↑	1.57	0.62
3^b^	5.26↓	0.14	0.02	0.07	0.07	0.22	0.07	0.68 ↑	2.17 ↑	0.39 ↑	0.3↑	38.75↑	22.14↑	0.99	0.54
4^a^	21.33	0.2	0.1	0.12	0.23	1.19 ↑	0.51↑	4.07 ↑	6.71 ↑	0.75 ↑	0.31↑	29.17↑	13.16↑	10.57↑	3.13 ↑
4^b^	4.12↓	0.12	0.02	0.04	0.06	0.21	0.02	0.47 ↑	1.44 ↑	0.31↑	0.35↑	24↑	72↑	0.89	0.71
Reference range	7–60	0.1–0.5	0–0.18	0–0.3	0–0.4	0–0.45	0–0.3	0–0.45	0–0.35	0–0.07	0–0.5	0.4–2.4	1–6	0.5–7.4	0.2–2.1

^a^ newborn screening.

^b^ reexamination.

### 3.2 Gene sequencing results

**FIGURE 1:**
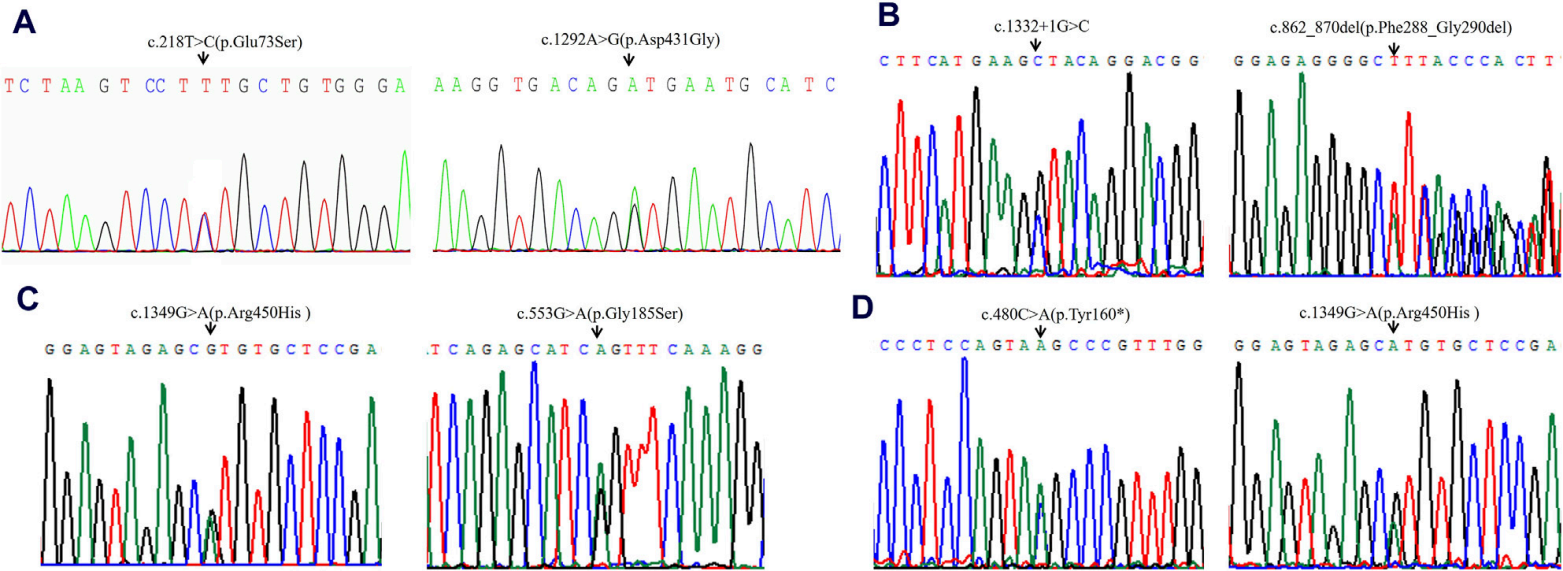
Sequencing analysis of all available members in four families with VLCADD. **(A)** In family 1, the patient’s parents were heterozygous carriers of c.218T>C (p.Glu73Ser) (paternal) and c.1292A>G (p.Asp431Gly) (maternal) in the *ACADVL* gene, respectively. **(B)** In family 2, the patient’s parents were heterozygous carriers of c.1332 + 1G>C (paternal) and c.862_870del (p.Phe288_Gly290del) (maternal) in the *ACADVL* gene, respectively. **(C)** In family 3, the patient’s parents were heterozygous carriers of c.1349G>A(p.Arg450His) (paternal) and c.553G>A (p.Gly185Ser) (maternal) in the *ACADVL* gene, respectively. **(D)** In family 4, the patient’s parents were heterozygous carriers of c.480C>A(p.Tyr160*) (paternal) and c.1349G>A (p.Arg450His) (maternal) in the *ACADVL* gene, respectively. Black arrows point to the mutation sites.

High-throughput sequencing revealed that case 1 carried compound heterozygous variants c.218T>C (p.Glu73Ser) and c.1292A>G (p.Asp431Gly) of the *ACADVL* gene (Figure 1A). Case 2 carried compound heterozygous variants c.1332 + 1G>C and c.862_870del (p.Phe288_Gly290del) of the *ACADVL* gene (Figure 1B). Case 3 carried compound heterozygous variants c.1349G>A (p.Arg450His) and c.553G>A (p.Gly185Ser) of the *ACADVL* gene (Figure 1C). Case 4 carried compound heterozygous variants c.480C>A (p.Tyr160*) and c.1349G>A (p.Arg450His) of the *ACADVL* gene (Figure 1D). Sanger sequencing confirmed that all four families followed an autosomal recessive inheritance pattern.

### 3.3 Pathogenicity prediction analysis

Variants c.218T>C (p.Glu73Ser), c.1332 + 1G>C, c.862_870del (p.Phe288_Gly290del), and c.480C>A (p.Tyr160*) are four novel variants that have not been reported (Table 1). These four novel variants had strong pathogenicity and were highly consistent with the patient’s clinical phenotype, which supports it as pathogenic evidence.

All missense variants indicated as pathogenic were analyzed using SIFT, PolyPhen-2, MutationTaster, and REVEL (Supplementary Table S3). Meanwhile, all the mutation-related diseases were highly consistent with the patient’s clinical phenotype, which was the supporting pathogenic evidence.

**FIGURE 2:**
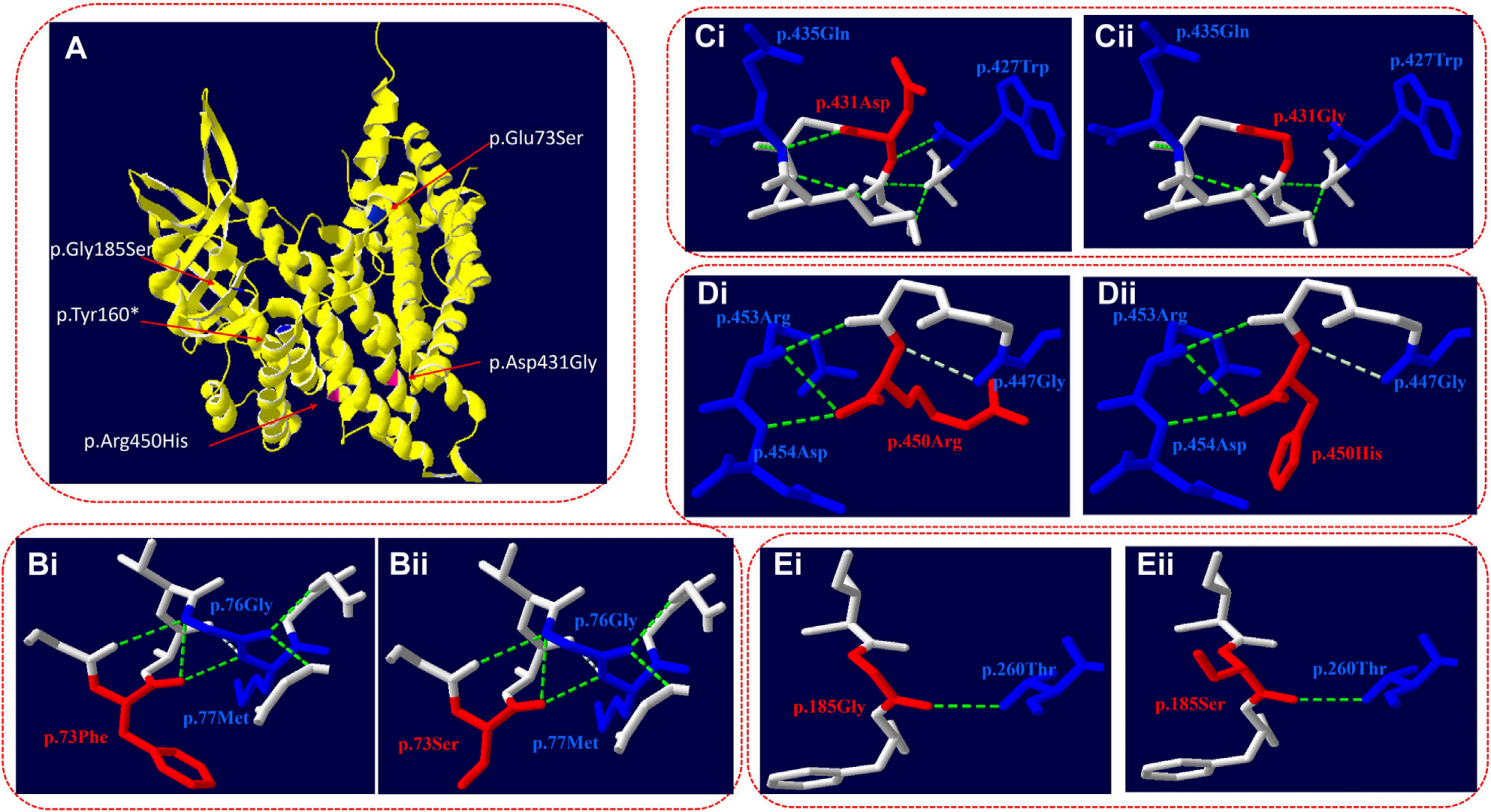
Three-dimensional structure of VLCAD (wild type and mutant). **(A)** Overall structure of the VLCAD protein. The chain of VLCAD is highlighted in yellow. The location of the missense mutations (p.Glu73Ser, p.Tyr 160*, and p.Gly185Ser) is mapped to the regions of the protein in blue. The location of the missense mutations (p.Asp431Gly and p.Arg450His) is mapped to the regions of the protein in red. **(B)** The p.73Phe missense mutation changes the structure of the side chain. **(C)** The p.431Asp missense mutation changes the hydrogen bond link between adjacent amino acids and the structure of the side chain. **(D)** The p.450Arg missense mutation changes the structure of the side chain. **(E)** The p.185Gly missense mutation changes the structure of the side chain.

Swiss-Pdb software was used to predict the effect of missense mutation on protein conformation in VLCAD (Figure 2A).

c.218T>C (p.Glu73Ser): in the three-dimensional structure of the human VLCAD protein, phenylalanine at position 73 forms hydrogen bonds with glycine at position 76 and methionine at position 77 (Figure 2Bi). The variant c.218T>C in the *ACADVL* gene resulted in non-polar phenylalanine at position 73 becoming polar serine. The structure of the side chain is changed (Figure 2Bii).

c.1292A>G (p.Asp431Gly): in the three-dimensional structure of the human VLCAD protein, aspartic acid at position 431 forms hydrogen bonds with tryptophan at position 427 and glutamine at position 435 (Figure 2Ci). The variant c.1292A>G in the *ACADVL* gene results in the substitution of aspartic acid at position 431 by glycine, which changes not only the hydrogen bond link between adjacent amino acids but also the structure of the side chain and the tertiary structure of the protein (Figure 2Cii).

c.1349G>A (p.Arg450His): in the three-dimensional structure of the human VLCAD protein, arginine at position 450 forms hydrogen bonds with glycine at position 447, arginine at position 453, and aspartic acid at position 454 (Figure 2Di). The variant c.1349G>A in the *ACADVL* gene causes arginine at position 450 to be replaced by histidine, which changes the structure of the side chain (Figure 2Dii).

c.553G>A (p.Gly185Ser): in the three-dimensional structure of the human VLCAD protein, glycine at position 185 forms hydrogen bonds with threonine at position 260 (Figure 2Ei). The variant c.553G>A in the *ACADVL* gene results in glycine at position 185 being replaced by serine, which changes the structure of the side chain (Figure 2Eii).

## 4 Discussion

Although fewer VLCADD cases are reported in China, the mortality rate is extremely high and seriously threatens the health of infants and children (Division of Biochemistry and Metabolism et al., 2022). Neonatal metabolic disease screening has included VLCADD as a major disease, and the disease can be effectively screened and diagnosed by tandem mass spectrometry combined with gene sequencing to achieve early screening, diagnosis, and treatment.

VLCADD is an autosomal recessive genetic disease. The pathogenic gene *ACADVL* (MIM 609575) is located on chromosome 17p13.1, containing 20 exons and 19 introns. The total length of the gene is 5.4 kb, encoding 655 amino acids. As of April 2023, 429 variants in the *ACADVL* gene have been included in the HGMD. There are various variants, including missense mutations, nonsense mutations, splicing mutations, and frameshift mutations; among them, missense mutations account for the largest proportion, accounting for 57.58% of the total variants, 20.28% of the frameshift variants, 11.66% of the splicing variants, and 10.48% of other variants.

In this study, four patients with VLCADD were diagnosed through high-throughput sequencing to identify their genetic causes. Seven kinds of variants were detected, with a total of eight variants, including four missense mutations, c.218T>C (p.Glu73Ser), c.1292A>G (p.D431G), c.1349G>A (p.Arg450His), and c.553G>A (p.Gly185Ser); one splicing mutation, c.1332 + 1G>C; one nonsense mutation, c.480C>A (p.Tyr160*); and one in-frame mutation, c.862_870del (p.Phe288_Gly290del). Four novel variants were detected. SWISS software was used to analyze the three-dimensional structure of the protein with incorrect meaning variation, and the results showed that c.218T>C (p.Glu73Ser), c.1349G>A (p.Arg450His), and c.553G>A (p.Gly185Ser) led to changes in the side chain structure (Figures 2B, D, E). The variant c.1292A>G (p.Asp431Gly) led to changes in the structure of connected hydrogen bonds and side chains (Figure 2C), which eventually led to changes in the three-dimensional structure of the protein.

Case 1 was a newborn with neonatal early-onset form and carried two missense variants, c.218T>C (p.Glu73Ser) and c.1292A>G (p.Asp431Gly) (Figure 1A). The patient appeared to have an acute metabolic crisis 3 months after birth and died in infancy (Table 1). The variant c.218T>C (p.Glu73Ser) is a novel variant; the adjacent location variation c.215C>T (p.Ser72Glu) has been reported (Zhang et al., 2014). The patient presented with symptoms at 5 months of age, including hepatomegalysis, hypotonia, growth retardation, and recurrent diarrhea. ECG analysis showed no apparent abnormality. He died of an acute infection at 8 months, and the patient’s sister died of similar symptoms when she was 6 months old. Genetic testing found that the patient carried compound heterozygous variants, Arg450His and Ser72Glu, and we hypothesized that both Ser72Glu and Glu73Ser might be associated with early neonatal onset. The variant c.1292A>G (p.Asp431Gly) is a reported variant (Bu and Pan, 2016). The proband in the report was screened by tandem mass spectrometry on day 14 after birth as vomiting and drowsiness had developed, which is early onset in newborns. Genetic testing revealed that the patient in the report carried 1843C>T (p.Arg615*) and c.1292A>G (p.Asp431Gly) compound heterozygous variants. From these two cases, we speculate that c.1292A>G (p.Asp431Gly) may be related to early onset in newborns.

Case 2 carried a compound heterozygous variant c.1332 + 1G>C and c.857_865del (p.Phe288_Gly290del) in the *ACADVL* gene (Figure 1B), developed acute metabolic crisis 2 days after birth, and died in the neonatal period (Table1). Splicing variant c.1332 + 1G>C leads to premature protein termination and loss of enzyme activity. Although the patient carried an in-frame variant, which will not lead to a severe condition, she was still onset and died during the neonatal period. We speculate that this may be because the 286–289 amino acids are located in the catalytic domain of the protein; the catalytic domain, the catalytic center of VLCAD proteins, is used to catalyze the synthesis of long-chain fatty acid β oxidation reaction and is the most important structural domain of VLCAD proteins. So, after the variant, it seriously affects the binding or catalytic function.

Case 3 carried the compound heterozygous variant c.1349G>A (p.Arg450His) and c.553G>A (p.Gly185Ser) of the *ACADVL* gene (Figure 1C). The enzyme activity of c.553G>A (p.Gly185Ser) is about 29% (Hoffmann et al., 2012). Two patients with the compound heterozygous variants c.553G>A (p.Gly185Ser)/c.878G>A (p.Gly294Glu) and c.553G>A (p.Gly185Ser)/753-2A>C (p.Asn252_His293del), respectively, had the phenotype of infantile form and neonatal early-onset form (Gobin-Limballe et al., 2010). The variant c.753-2A>C (p.Asn252_His293del) is a common variant in Italian VLCADD patients, and incorrect splicing leads to the degradation of VLCAD mRNA, so we infer that c.553G>A (p.Gly185Ser) should not cause a severe phenotype.

The variant c.1349G>A (p.Arg450His) has numerous case reports. A patient was diagnosed with VLCADD 33 days after birth and recurrent rhabdomyolysis at 11 months. At present, following the patient up to 5.8 years of age with no other symptoms mentioned in the literature showed that the patient might have a relatively good prognosis. The patient carries a homozygous variant of c.1349G>A (p.Arg450His) (Kang et al., 2018). A 14-year-old Japanese girl presented with recurrent myalgia with elevated serum creatine kinase levels after moderate exercise. She was clinically diagnosed with myopathy-type VLCADD (Fukao et al., 2001). This patient first presented with clinical symptoms at 6 years of age and did not present with hypoglycemic liver enlargement and cardiomyopathy. Sequencing revealed that the patient had a compound heterozygous variant in the *ACADVL* gene Ala416Thr and Arg450His. The enzyme activity of the Ala416Thr mutant was 10%–20% of that of the wild type, and the Arg450His mutant was 0%–5% of that of the wild type. Arg450His showed 5% normal-to-low residual VLCAD activity at 30°C; however, the p.Arg450His variant had no significant activity at 37°C, indicating that the homozygous p.Arg450His variant is a mild variant sensitive to temperature *in vitro*, which may be associated with the milder phenotype of VLCADD (Fukao et al., 2001; Li et al., 2020). Among the six VLCADD patients treated at Peking University First Hospital, three were found to carry Arg450His variants, which may be a high-frequency variant in the Chinese population (Li et al., 2020).

In this study, cases 3 and 4 carried a variant of c.1349G>A (p.Arg450His). According to the genotype and phenotypic correlation analysis, although cases 3 and 4 were predicted to be late-onset form, case 4 carried the nonsense variant c.480C>A (p.Tyr160*), so the phenotype of the child should be closely monitored.

There was a significant correlation between the genotype and phenotype of VLCADD. The nonsense variant of the *ACADVL* gene can cause the complete loss of enzyme activity, resulting in severe clinical phenotypes. The occurrence of deletions or insertions and splicing variants will cause premature termination of the protein. The result is similar to that of a nonsense variant, which can cause a severe cardiomyopathy type. In the literature, 81% of truncated variants (nonsense and splicing) are associated with the cardiomyopathy type (Andresen et al., 1999; Miller et al., 2015). However, a missense variant or in-frame variant can leave some residual enzyme activity, with mild symptoms. Moreover, 82% of hepatopathy type and 93% of myopathy type are missense variants or in-frame variants (Spiekerkoetter et al., 2009). Some studies have shown that certain variants in the gene are associated with phenotypes. The variants c.848T>C (p.Val283Ala), c.227G>A (p.Arg76Glu), and c.1349G>A (p.Arg450His) are considered to be associated with mild VLCADD. The variants c.1405C>T (p.Arg469Trp) and c.1358G>A (p.Arg53Gln) may be associated with severe VLCADD (Andresen et al., 1999; Spiekerkoetter et al., 2009; Miller et al., 2015; Fukao et al., 2001; Miller et al., 2015; Li et al., 2020).

Based on literature reports, it is possible to conduct an association analysis between the genotype and phenotype, but it cannot be too simplistic to directly use the genotype to determine the clinical phenotype. A study has found that siblings carrying the same variant may also have different onset times and symptoms, indicating that it is difficult to accurately predict the clinical disease course and outcome using genetic variants (Watanabe et al., 2018). Meanwhile, the enzyme activity is not only related to the type of gene variant but also may be affected by external conditions such as temperature, which can only be used for reference analysis. Even if a patient carries two missense variants, it does not indicate a good prognosis (Gregersen et al., 2001; Pena et al., 2016). Two of the cases in this group died, and all four alleles were missense variants. Therefore, the prognosis could not be judged by the genotype alone. At the same time, our sample size is relatively small, so it is necessary to establish a Chinese VLCADD cohort database, which is more instructive for the analysis of the genotypic and phenotypic correlations.

Although this disease can be screened early, for early-onset children, early onset carries a high risk of death in the neonatal period. Therefore, early screening is particularly important for disease screening and diagnosis. Tandem mass spectrometry detection is essential for diagnosing VLCADD, and an elevation in C14:1 levels is the most sensitive indicator of VLCADD. Meanwhile, the sensitivity and specificity of C14:1/C12:1 are better than that of C14:1 and other acylcarnitine ratios, which is more valuable for the early detection of disease (Division of Biochemistry and Metabolism et al., 2022). Gene diagnosis can be carried out in time for children with C14:1 increase but less than 1 umol/L in neonatal screening (Bleeker et al., 2019; Wood et al., 2001). High-throughput sequencing technology is used to explore the genetic etiology of VLCADD and establish the correlation between the genotype and phenotype, which is crucial for the rapid diagnosis of disease, early symptomatic intervention, and prenatal testing.

## 5 Conclusion

In conclusion, a detailed analysis of the clinical conditions and gene variants of four cases of VLCADD was conducted in this study, four novel pathogenic variants of the *ACADVL* gene were found through genetic testing, the *ACADVL* gene mutation spectrum was enriched, and the correlation between the genotype and phenotype of four cases was analyzed. VLCADD is a preventable and curable genetic metabolic disease, and the prognosis varies among different types of patients. In this study, two patients had cardiomyopathy type and died during infancy and the neonatal period, respectively. Therefore, early identification of the cardiomyopathy type is necessary.

This study suggests that clinicians should pay more attention to genetic testing, use high-throughput sequencing technology to quickly identify the cause of disease, and enhance the efficiency of disease diagnosis. Clinical trials should actively carry out neonatal screening and early diagnosis so that VLCADD patients can receive timely and effective treatment and improve the survival rate and quality of life of children.

## Data Availability

The original contributions presented in the study are included in the article/Supplementary Material; further inquiries can be directed to the corresponding authors.

## Abbreviations

VLCADD, very-long-chain acyl-CoA dehydrogenase deficiency; MS/MS, tandem mass spectrometry; GC/MS, gas chromatography; VLCAD, very-long-chain acyl-CoA dehydrogenase; HGMD, Human Gene Mutation Database; UCSC, University Of California Santa Cruz; SIFT, Sorting Intolerant From Tolerant

## References

[B1] Al Bandari M. Nagy L. Cruz V. Hewson S. Hossain A. Inbar-Feigenberg M. (2024). Management and outcomes of very long-chain acyl-CoA dehydrogenase deficiency (VLCAD deficiency): a retrospective chart review. IJNS 10, 29. 10.3390/ijns10020029 38651394 PMC11036265

[B2] Andresen B. S. Olpin S. Poorthuis B. J. Scholte H. R. Vianey-Saban C. Wanders R. (1999). Clear correlation of genotype with disease phenotype in very-long-chain acyl-CoA dehydrogenase deficiency. Am. J. Hum. Genet. 64, 479–494. 10.1086/302261 9973285 PMC1377757

[B3] Aoyama T. Uchida Y. Kelley R. I. Marble M. Hofman K. Tonsgard J. H. (1993). A novel disease with deficiency of mitochondrial very-long-chain acyl-CoA dehydrogenase. Biochem. Biophysical Res. Commun. 191, 1369–1372. 10.1006/bbrc.1993.1368 8466512

[B4] Arnold G. L. Van Hove J. Freedenberg D. Strauss A. Longo N. Burton B. (2009). A Delphi clinical practice protocol for the management of very long chain acyl-CoA dehydrogenase deficiency. Mol. Genet. Metab. 96, 85–90. 10.1016/j.ymgme.2008.09.008 19157942 PMC3219055

[B5] Bertrand C. Largillière C. Zabot M. T. Mathieu M. Vianey-Saban C. (1993). Very long chain acyl-CoA dehydrogenase deficiency: identification of a new inborn error of mitochondrial fatty acid oxidation in fibroblasts. Biochimica Biophysica Acta (BBA) - Mol. Basis Dis. 1180, 327–329. 10.1016/0925-4439(93)90058-9 8422439

[B6] Bleeker J. C. Kok I. L. Ferdinandusse S. Van Der Pol W. L. Cuppen I. Bosch A. M. (2019). Impact of newborn screening for very‐long‐chain acyl‐CoA dehydrogenase deficiency on genetic, enzymatic, and clinical outcomes. J. Inherit. Metab. Dis., jimd12075. 10.1002/jimd.12075 30761551

[B7] Boneh A. Andresen B. S. Gregersen N. Ibrahim M. Tzanakos N. Peters H. (2006). VLCAD deficiency: pitfalls in newborn screening and confirmation of diagnosis by mutation analysis. Mol. Genet. Metabolism 88, 166–170. 10.1016/j.ymgme.2005.12.012 16488171

[B8] Bu Q. Pan Z. (2016). A novel missense mutation in very long-chain acyl-CoA dehydrogenase deficiency. Indian Pediatr. 53, 262. 10.1007/s13312-016-0833-0 27029698

[B9] Burrage L. C. Miller M. J. Wong L.-J. Kennedy A. D. Sutton V. R. Sun Q. (2016). Elevations of C14:1 and C14:2 plasma acylcarnitines in fasted children: a diagnostic dilemma. J. Pediatr. 169, 208–213. 10.1016/j.jpeds.2015.10.045 26602010 PMC4729603

[B10] Division of Biochemistry and Metabolism, Medical Genetics Branch, Chinese Medical Association, Division of Genetics and Metabolism, Child Diseases and Health Care Branch, Chinese Association for Maternal, and Child Health (2022). Expert consensus on diagnosis and treatment of very long-chain acyl-CoA dehydrogenase deficiency. Zhejiang Da Xue Xue Bao Yi Xue Ban. 51, 122–128. 10.3724/zdxbyxb-2022-0107 36161784 PMC9109756

[B11] El-Gharbawy A. Vockley J. (2018). Inborn errors of metabolism with myopathy: defects of fatty acid oxidation and the carnitine shuttle system. Pediatr. Clin. North Am. 65, 317–335. 10.1016/j.pcl.2017.11.006 29502916 PMC6566095

[B12] Fukao T. Watanabe H. Orii K. Takahashi Y. Hirano A. Kondo T. (2001). Myopathic form of very-long chain acyl-coa dehydrogenase deficiency: evidence for temperature-sensitive mild mutations in both mutant alleles in a Japanese girl. Pediatr. Res. 49, 227–231. 10.1203/00006450-200102000-00016 11158518

[B13] Gobin-Limballe S. McAndrew R. P. Djouadi F. Kim J.-J. Bastin J. (2010). Compared effects of missense mutations in Very-Long-Chain Acyl-CoA Dehydrogenase deficiency: combined analysis by structural, functional and pharmacological approaches. Biochimica Biophysica Acta (BBA) - Mol. Basis Dis. 1802, 478–484. 10.1016/j.bbadis.2010.01.001 PMC3401415 20060901

[B14] Gregersen N. Andresen B. S. Corydon M. J. Corydon T. J. Olsen R. K. Bolund L. (2001). Mutation analysis in mitochondrial fatty acid oxidation defects: exemplified by acyl-CoA dehydrogenase deficiencies, with special focus on genotype-phenotype relationship. Hum. Mutat. 18, 169–189. 10.1002/humu.1174 11524729

[B15] Hoffmann L. Haussmann U. Mueller M. Spiekerkoetter U. (2012). VLCAD enzyme activity determinations in newborns identified by screening: a valuable tool for risk assessment. J. Inherit. Metab. Dis. 35, 269–277. 10.1007/s10545-011-9391-8 21932095

[B16] Kang E. Kim Y.-M. Kang M. Heo S.-H. Kim G.-H. Choi I.-H. (2018). Clinical and genetic characteristics of patients with fatty acid oxidation disorders identified by newborn screening. BMC Pediatr. 18, 103. 10.1186/s12887-018-1069-z 29519241 PMC5842515

[B17] Li X. Ma R. Liu Y. Kang L. He R. Song J. (2020). One potential hotspot ACADVL mutation in Chinese patients with very-long-chain acyl-coenzyme A dehydrogenase deficiency. Clin. Chim. Acta 503, 218–222. 10.1016/j.cca.2019.11.034 31794763

[B18] Miller M. J. Burrage L. C. Gibson J. B. Strenk M. E. Lose E. J. Bick D. P. (2015). Recurrent ACADVL molecular findings in individuals with a positive newborn screen for very long chain acyl-coA dehydrogenase (VLCAD) deficiency in the United States. Mol. Genet. Metabolism 116, 139–145. 10.1016/j.ymgme.2015.08.011 PMC4790081 26385305

[B19] Nurjanah S. Gerding A. Vieira-Lara M. A. Evers B. Langelaar-Makkinje M. Spiekerkoetter U. (2023). Heptanoate improves compensatory mechanism of glucose homeostasis in mitochondrial long-chain fatty acid oxidation defect. Nutrients 15, 4689. 10.3390/nu15214689 37960342 PMC10649308

[B20] Pena L. D. M. van Calcar S. C. Hansen J. Edick M. J. Walsh Vockley C. Leslie N. (2016). Outcomes and genotype-phenotype correlations in 52 individuals with VLCAD deficiency diagnosed by NBS and enrolled in the IBEM-IS database. Mol. Genet. Metabolism 118, 272–281. 10.1016/j.ymgme.2016.05.007 PMC4970910 27209629

[B21] Rovelli V. Manzoni F. Viau K. Pasquali M. Longo N. (2019). Clinical and biochemical outcome of patients with very long-chain acyl-CoA dehydrogenase deficiency. Mol. Genet. Metab. 127, 64–73. 10.1016/j.ymgme.2019.04.001 31031081

[B22] Schymik I. Liebig M. Mueller M. Wendel U. Mayatepek E. Strauss A. W. (2006). Pitfalls of neonatal screening for very-long-chain acyl-CoA dehydrogenase deficiency using tandem mass spectrometry. J. Pediatr. 149, 128–130. 10.1016/j.jpeds.2006.02.037 16860141

[B23] Shibata N. Hasegawa Y. Yamada K. Kobayashi H. Purevsuren J. Yang Y. (2018). Diversity in the incidence and spectrum of organic acidemias, fatty acid oxidation disorders, and amino acid disorders in Asian countries: selective screening vs. expanded newborn screening. Mol. Genet. Metab. Rep. 16, 5–10. 10.1016/j.ymgmr.2018.05.003 29946514 PMC6014585

[B24] Spiekerkoetter U. Lindner M. Santer R. Grotzke M. Baumgartner M. R. Boehles H. (2009). Management and outcome in 75 individuals with long-chain fatty acid oxidation defects: results from a workshop. J. Inherit. Metab. Dis. 32, 488–497. 10.1007/s10545-009-1125-9 19399638

[B25] Strauss A. W. Powell C. K. Hale D. E. Anderson M. M. Ahuja A. Brackett J. C. (1995). Molecular basis of human mitochondrial very-long-chain acyl-CoA dehydrogenase deficiency causing cardiomyopathy and sudden death in childhood. Proc. Natl. Acad. Sci. U. S. A. 92, 10496–10500. 10.1073/pnas.92.23.10496 7479827 PMC40638

[B26] Vianey-Saban C. Divry P. Brivet M. Nada M. Zabot M. T. Mathieu M. (1998). Mitochondrial very-long-chain acyl-coenzyme A dehydrogenase deficiency: clinical characteristics and diagnostic considerations in 30 patients. Clin. Chim. Acta 269, 43–62. 10.1016/s0009-8981(97)00185-x 9498103

[B27] Watanabe K. Yamada K. Sameshima K. Yamaguchi S. (2018). Two siblings with very long-chain acyl-CoA dehydrogenase (VLCAD) deficiency suffered from rhabdomyolysis after l-carnitine supplementation. Mol. Genet. Metab. Rep. 15, 121–123. 10.1016/j.ymgmr.2018.03.007 30023301 PMC6047112

[B28] Wood J. C. Magera M. J. Rinaldo P. Seashore M. R. Strauss A. W. Friedman A. (2001). Diagnosis of very long chain acyl-dehydrogenase deficiency from an infant’s newborn screening card. Pediatrics 108, E19. 10.1542/peds.108.1.e19 11433098

[B29] Zhang R.-N. Li Y.-F. Qiu W.-J. Ye J. Han L.-S. Zhang H.-W. (2014). Clinical features and mutations in seven Chinese patients with very long chain acyl-CoA dehydrogenase deficiency. World J. Pediatr. WJP 10, 119–125. 10.1007/s12519-014-0480-2 24801231

[B30] Zytkovicz T. H. Fitzgerald E. F. Marsden D. Larson C. A. Shih V. E. Johnson D. M. (2001). Tandem mass spectrometric analysis for amino, organic, and fatty acid disorders in newborn dried blood spots: a two-year summary from the New England Newborn Screening Program. Clin. Chem. 47, 1945–1955. 10.1093/clinchem/47.11.1945 11673361

