# Supplementary material for: Four novel variants identified in the ACADVL gene causing very-long-chain acyl-coenzyme A dehydrogenase deficiency in four unrelated Chinese families
Source: Front Genet. 2024 Aug 12;15:1433160. doi: 10.3389/fgene.2024.1433160 (PMC11345273; doi:10.3389/fgene.2024.1433160)
Supplement: Supplementary file 1 [file Table1.DOCX]

**Supplementary Table 1. Primers used in this study.**

| **Primer name** | **Sequence (5' → 3')** | **T_m_ (°C)** | **Size (bp)** |
| --- | --- | --- | --- |
| *ACADVL-*F1 | TCCCTTCCCTGAACTTGCTA | 60 | 362 |
| *ACADVL-*R1 | CTGTGTCTGCTCTTCGTTGA |  |  |
| *ACADVL-*F2 | GCCCACACTCTCCTGTTAAG | 60 | 964 |
| *ACADVL-*R2 | AAAGGCCACATTCAGTCCAC |  |  |
| *ACADVL-*F3 | GAGTGCTAACATGGACCAGG | 60 | 414 |
| *ACADVL-*R3 | CTTACCATACAGCCCTGCAG |  |  |

**Supplementary Table 2. Test results of gas chromatography-mass spectrometry**

| Case | **Gas chromatography-mass spectrometry** | | | | | | | |
| --- | --- | --- | --- | --- | --- | --- | --- | --- |
|  | Glutaric acid | Adipic acid | Octametic acid | Azelaic acid | Lactic acid | Pimelic acid | 2-OH-sebacic acid | 2-OH-adipic acid |
| 3 | 2.88 | 6.4 ↑ | 4.82 ↑ | 2.99 | 3.01 | 6.26 | 1.05 | 2.71 ↑ |
| 4 | 8.67 ↑ | 8.86 ↑ | 8.1 ↑ | 19.41 ↑ | 10.69 ↑ | 24.36 ↑ | 8.08 ↑ | 6.2 ↑ |
| Reference range | 0-4 | 0.5-5 | 0.3-4.7 | 0-10.7 | 0-4.7 | 0-9.3 | 0-5.3 | 0-2 |

**Supplementary Table 3. Bioinfomatic analysis of missense variants**

|  | **ExAC ALL** | **GnomAD EAS** | **GnomAD** **ALL** | **SIFT** | **Polyphen2** | **MutationTaster** | **REVEL** |
| --- | --- | --- | --- | --- | --- | --- | --- |
| c.218T>C | - | - | - | D(0) | D(0.996) | D(1) | D(0.883) |
| c.1292A>G | - | - | - | D(0.027) | B(0.407) | D(1) | D(0.892) |
| c.1349G>A | 0.00004123 | 0.00016311 | 0.00002387 | D (0.001) | D(0.989) | D(1) | D(0.92) |
| c.553G>A | 0.00001649 | 0.00001193 | - | D (0) | D(1) | D(1) | D(0.971) |

D(Damaging);B(Benign);P(Pathogenic); LP(Likely pathogenic);VUS(Uncertain significance)
